# Dealing with missing phase and missing data in phylogeny-based analysis

**DOI:** 10.1186/1753-6561-1-s1-s22

**Published:** 2007-12-18

**Authors:** Claire Bardel, Pascal Croiseau, Emmanuelle Génin

**Affiliations:** 1 UMR 5145 – Génétique des Populations Humaines – CNRS MNH, Université Paris VII, 17 Place du Trocadero, Paris, 75016 France; 2 INSERM U535, BP 1000, Villejuif, 94817 France; 3 Universite Paris-Sud UMR-S535, Villejuif, 94817 France

## Abstract

We recently described a new method to identify disease susceptibility loci, based on the analysis of the evolutionary relationships between haplotypes of cases and controls. However, haplotypes are often unknown and the problem of phase inference is even more crucial when there are missing data. In this work, we suggest using a multiple imputation algorithm to deal with missing phase and missing data, prior to a phylogeny-based analysis. We used the simulated data of Genetic Analysis Workshop 15 (Problem 3, answer known) to assess the power of the phylogeny-based analysis to detect disease susceptibility loci after reconstruction of haplotypes by a multiple-imputation method. We compare, for various rates of missing data, the performance of the multiple imputation method with the performance achieved when considering only the most probable haplotypic configurations or the true phase. When only the phase is unknown, all methods perform approximately the same to identify disease susceptibility sites. In the presence of missing data however, the detection of disease susceptibility sites is significantly better when reconstructing haplotypes by multiple imputation than when considering only the best haplotype configurations.

## Background

In the last few years, various phylogeny-based approaches have been developed to test for association between a candidate gene and a disease [1-4]. These tests are based on the grouping of haplotypes according to their evolutionary relationships represented by a phylogenetic tree. This grouping reduces the degree of freedom of the association tests and thus, increases their power. Interestingly, the haplotype phylogeny can also be used to precisely identify loci involved in the determinism of the disease. We recently described a new method to localize disease susceptibility loci (DS loci), based on the definition of a co-evolution index (*V*_*i*_) between the markers and the disease. The markers showing the highest *V*_*i *_are assumed to be putative DS sites [4]. Simulations have shown that the method performs well at identifying DS loci, especially when several DS loci exist.

To reconstruct the phylogenetic tree, haplotype information is used. In most situation, this information is not available from the data and needs to be inferred. In our method, this was done by determining the most probable haplotypes of the different individuals and analyzing them as if they were the known haplotypes. However, this approach may lead to incorrect inferences because it does not take into account the uncertainty of the phase that might be very large, especially in the presence of missing data. In this context, the use of multiple imputation to reconstruct missing phase and missing data might be an interesting alternative. In this paper, we used the simulated data of Genetic Analysis Workshop 15 (GAW15) to compare the relative power of these two approaches to haplotype reconstruction to correctly identify the simulated DS sites when using a phylogeny-based analysis.

## Methods

### Data

We analyzed the 100 replicates simulated for GAW 15 (Problem 3). To apply a phylogeny-based method, we need to work on a candidate region where the disease susceptibility site is typed, and where the recombination rate is low. We used the answers to choose a 200-kb region of chromosome 6 around the DR locus that contained two DS sites: the DR locus and locus C. In this region, nine single-nucleotide polymorphisms (SNPs) (including locus C) were selected. A tenth biallelic locus was added, corresponding to the DR locus in which the lower risk alleles DR1 and DRX were pooled. The linkage disequilibrium is low within these ten sites: the highest *r*^2 ^is between locus C and SNP 4 (*r*^2 ^= 0.65) and it is the only pair of loci with an *r*^2 ^above 0.2.

For each replicate the first affected child of the first 500 families was selected to obtain 500 trios. Missing data were generated on the different loci (with the same percentage of missing data on each locus) on both parents and children. In each replicates, the same individuals had their genotypes missing at the same loci in order to ensure a similar pattern of missing data over replicates.

### Reconstruction of missing data and missing phases

Missing phases and missing genotypes were reconstructed either only by an algorithm to infer the most probable haplotypes without missing data for each individual, or by a multiple imputation method. For both methods, the first step was the inference of all the possible haplotypic configurations and their probabilities. It was performed with the software ZAPLO [5]. The first method then consists of picking the most likely haplotypes for each individual. The only families kept for the analysis were those with a low level of haplotype uncertainty; i.e., families with a best configuration posterior probability >50% and at least 25% difference between the posterior probabilities of the best and second best configuration. Similar results were obtained with other cut-off values (data not shown). The multiple imputation procedure is the same as the one described in Croiseau et al. [6]. Briefly, it consists of repeating two steps: 1) given the current values of two parameters (population haplotype frequencies and affected child genotype frequencies), sampling a complete data set according to the posterior probabilities of each genotypic configuration and 2) given the current data set, updating the two parameters. After a burn-in period of 1000 iterations, every 1000 iterations, the current complete data file was retained. We ran the algorithm until we obtained ten complete data sets.

### Identification of the susceptibility sites

The identification of the DS sites was performed with the software ALTree [7]. At first, 1000 equiparsimonious unrooted trees were reconstructed for the 30 most frequent haplotypes using the parsimony method implemented in the software PAUP*, version 4.0b10 [8]. To ensure that various tree configurations were explored, PAUP* was launched 10 times, 100 trees being retained each time. Then, a new character called *S*, which represents the disease status, was defined for each haplotypes. The state of this character depends on the proportion of cases carrying a given haplotype (state 1 for a large proportion of cases and 0 otherwise). The character state changes were optimized on the tree for each character (including *S*) using the deltran option. A correlated evolution index (*V*_*i*_) was calculated between the changes of each site *i *and the changes of the character *S*. This index was defined as the difference between the number of observed and expected co-mutations between site *i *and character *S*, divided by the square root of the number of expected co-mutations [4]. To take into account the 10 imputed data sets, we calculated the median of the *V*_*i *_over these 10 data sets. Finally, the sites with *V*_*i *_≤ 0 were discarded and the two site(s) with the highest *V*_*i *_are retained as putative DS sites.

## Results

The power to identify the DS sites is measured as the percentage of replicates among the 100 replicates available in which the simulated DS sites have the highest *V*_*i*_.

### Missing phase

In Table 1, we compare the power to identify the DS sites on the complete data set in three conditions: 1) when the phase is known, 2) when only the best haplotype configuration is kept, or 3) when using multiple imputation to infer missing data. The results show no significant difference between the three methods.

**Table 1:** Power to identify the true susceptibility loci using different methods to infer phases

	% Power (95% confidence interval)		
	
Method	Phased^a^	Most likely haplotypes^b^	Imputation^c^
2 sites	81 (73.3–88.7)	84 (76.8–91.2)	78 (69.9–86.1)
1 site only^d^	12 (5.6–18.4)	8 (2.7–13.3)	13 (6.4–19.6)
1 site + 1 error	7 (2.0–12.0)	8 (2.7–13.3)	9 (3.4–14.6)

^a ^The phase given in the data is used.

^b ^The phase is inferred using ZAPLO (selection of the most likely haplotypes).

^c ^The phase is inferred using ZAPLO and multiple imputation.

^d ^Only one site has a *V*_*i *_> 0 and it is a true DS site.

### Missing data

**Figure 1:**
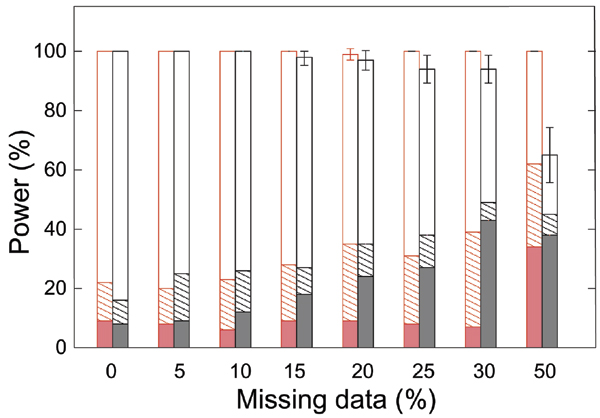
**Power to identify one of the two susceptibility sites for different rates of missing data**. Missing data and missing phases are reconstructed using a multiple imputation method (in red) or the most likely haplotypes obtained with ZAPLO (in black). The percentage of replicates in which the site with the highest V_i _is one of the simulated DS sites is shown according to the properties of the second-best site (if any): i) no second-best site is identified with a *V*_*i *_> 0 (striped bars); ii) the second-best site is a DS site (open bars); iii) the second-best site is not a DS site (colored bars).

Figure 1 shows that for different rates of missing data, the percentage of replicates in which the site with the highest *V*_*i *_(best site) is one of the two true DS site. Interestingly, this percentage remains very similar for the different rates of missing data when using multiple imputation. This is not the case when considering only the most likely haplotypes with more than 15% of missing data. With the multiple imputation, there are fewer errors on the second best site and more replicates in which no other site is detected than when using the most likely haplotypes (Figure 1).

**Figure 2:**
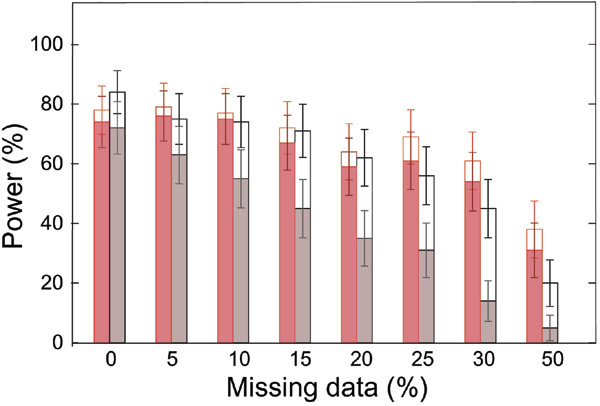
**Power to identify the two susceptibility sites for different rates of missing data**. Missing data and missing phases are reconstructed using a multiple imputation method (in red) or the most likely haplotypes obtained with ZAPLO (in black). The percentage of replicates in which the two sites with the highest V_i _values are DR and locus C are reported in the two situations in which there are other sites with *V*_*i *_> 0 (open bars) or there is no other site with *V*_*i *_> 0 (colored bars).

Figure 2 shows the percentage of replicates in which the two best sites are the two simulated DS sites. For up to 20% of missing data, there is no difference of power when using the most likely haplotypes or the multiple imputation method. For higher rates of missing data, the multiple imputation method leads to higher power but this is not significant at the 5% level because the 95% confidence intervals overlap. The multiple imputation method is found to be more accurate: it is significantly more powerful to identify only the two DS sites (no other site having a *V*_*i *_> 0).

**Figure 3:**
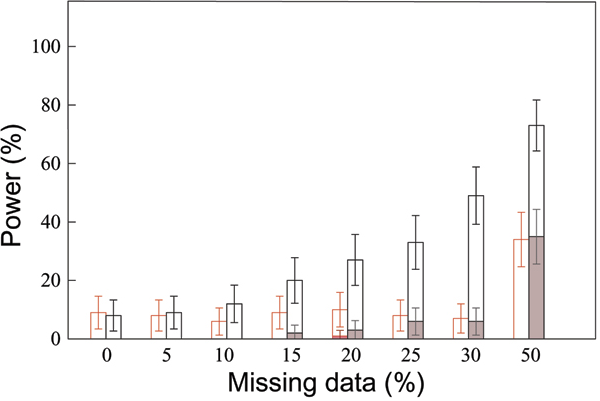
**Error in the identification of the susceptibilityloci for different rates of missing data**. Missing data and missing phases are reconstructed using a multiple imputation method (in red) or the most likely haplotypes obtained with ZAPLO (in black). Colored bars: the best site (with the highest *V*_*i*_) is neither DR nor locus C. Empty bars: sum of two error rates, error on the best site and error on the second best site only (i.e., the site with the highest V_i _is either locus C or DR, but the site with the second highest *V*_*i *_is neither locus C nor DR).

The error rate, defined as the percentage of replicates in which the true DS sites are not correctly identified, is presented in Figure 3. With the multiple imputation method, the locus with the highest *V*_*i *_is always either DR or locus C (more often DR). There is also significantly less error on the two best sites (sum of the error on the best site and on the second best site) than when the most likely haplotypes are used. Indeed, this two best site error rate is stable at around 10% for up to 30% of missing data and increases to 35% for 50% missing data. On the contrary, when the most likely haplotypes are used, the two best site error rate constantly increases and reaches 70% for 50% of missing data.

## Discussion

The analysis of the GAW15 simulated data allowed us to confirm the power of phylogeny-based tests to identify several DS sites located in the same region. We have shown that the method is particularly powerful to identify locus DR as a susceptibility site. This may be explained by the very high risks attributed to individuals carrying the DR4 allele. The method also allowed us to detect locus C, generally as the second best site and with a lower power than DR (Figure 3 shows more errors on the second best site than on the best site). However, this locus only increases the risk in women, and our analysis has been performed regardless of the sex of the individuals.

Our results show that the use of a multiple imputation method to reconstruct haplotypes allows a better detection of the DS sites in the presence of missing data than the use of the best haplotypic configuration. In particular, it is more accurate (the DS sites are often the only one detected) and it drastically decreases the error rate for the DS site identification. In this study, in the absence of missing data, no difference between the three phase imputation methods was found, but this is probably a particular situation where phase is not very ambiguous thanks to the familial information available. Indeed, when we use the most likely haplotypes, only a mean of 12.48 families (over 500 families in the sample) are discarded from the analysis because of their high level of phase uncertainty. The relative performance of the three methods might be different using case-control data with no familial information available.

To tackle the problem of phase resolution, two types of strategies were suggested. In one-stage procedures, the phase inference and the analysis are performed simultaneously. In two-stage procedures, haplotype frequencies that are estimated in the first stage are used as weights in the second stage. Concerning phylogeny-based analyses, a one-stage procedure will be very difficult to develop because haplotypes need to be known to reconstruct the phylogenetic tree. This probably explains why only two-stage procedures have been proposed [2,9]. The problem with these different two-stage methods is that the phylogenetic tree is reconstructed on all possible haplotypes, even if they do not really exist. This can significantly increase the number of haplotypes considered, and thus, lead to an increase in the computation time (especially for parsimony-based tree reconstruction) and possibly, to a loss in power. With the multiple imputation method, ten imputed files are analyzed, which will also increase the computation time, but only the haplotypes observed in these files are used in the phylogenetic reconstruction. Further work will need to be done to compare the multiple imputation approach with these two-stage procedures.

## Conclusion

In conclusion, the analysis of the GAW15 simulated data shows that multiple imputation can be of great value in dealing with missing genotypes prior to a phylogeny-based analysis. In comparison with a strategy using only the most likely haplotypes, it increases the chances to correctly identify disease susceptibility loci.

## Competing interests

The author(s) declare that they have no competing interests.
